# A Comparison of the Whole Genome Approach of MeDIP-Seq to the Targeted Approach of the Infinium HumanMethylation450 BeadChip^®^ for Methylome Profiling

**DOI:** 10.1371/journal.pone.0050233

**Published:** 2012-11-29

**Authors:** Christine Clark, Priit Palta, Christopher J. Joyce, Carol Scott, Elin Grundberg, Panos Deloukas, Aarno Palotie, Alison J. Coffey

**Affiliations:** 1 Department of Human Genetics, Wellcome Trust Sanger Institute, Wellcome Trust Genome Campus, Hinxton, Cambridge, United Kingdom; 2 Department of Bioinformatics, Institute of Molecular and Cell Biology, University of Tartu, Tartu, Estonia; 3 Institute for Molecular Medicine Finland (FIMM), University of Helsinki, Helsinki, Finland; 4 Program in Medical and Population Genetics and Genetic Analysis Platform, The Broad Institute of MIT and Harvard, Cambridge, Massachusetts, United States of America; 5 Department of Medical Genetics, University of Helsinki and University Central Hospital, Helsinki, Finland; Bellvitge Biomedical Research Institute (IDIBELL), Spain

## Abstract

DNA methylation is one of the most studied epigenetic marks in the human genome, with the result that the desire to map the human methylome has driven the development of several methods to map DNA methylation on a genomic scale. Our study presents the first comparison of two of these techniques - the targeted approach of the Infinium HumanMethylation450 BeadChip® with the immunoprecipitation and sequencing-based method, MeDIP-seq. Both methods were initially validated with respect to bisulfite sequencing as the gold standard and then assessed in terms of coverage, resolution and accuracy. The regions of the methylome that can be assayed by both methods and those that can only be assayed by one method were determined and the discovery of differentially methylated regions (DMRs) by both techniques was examined. Our results show that the Infinium HumanMethylation450 BeadChip® and MeDIP-seq show a good positive correlation (Spearman correlation of 0.68) on a genome-wide scale and can both be used successfully to determine differentially methylated loci in RefSeq genes, CpG islands, shores and shelves. MeDIP-seq however, allows a wider interrogation of methylated regions of the human genome, including thousands of non-RefSeq genes and repetitive elements, all of which may be of importance in disease. In our study MeDIP-seq allowed the detection of 15,709 differentially methylated regions, nearly twice as many as the array-based method (8070), which may result in a more comprehensive study of the methylome.

## Introduction

DNA methylation is one of the most studied epigenetic marks in the human genome involving the covalent addition of a methyl group to the fifth carbon of cytosine residues predominantly within the context of CpG dinucleotides. Patterns of DNA methylation are determined in early development [1], are heritable [2] and stably maintained through cell division but can also be dynamic in response to environment [3]. Changes in methylation patterns are an essential mechanism used to control many biological processes including gene regulation, X chromosome inactivation, genomic imprinting and cellular differentiation. There is an ever increasing list of diseases, including a wide variety of cancers [4], in which alterations in DNA methylation patterns can be demonstrated to be either a causal factor in, or a consequence of disease [5], [6]. A complete characterisation of the methylome and the dynamic changes that occur within it may in some cases serve as an accurate predictor of prognosis and treatment success [7].

The desire to map the entire methylome has driven the development of large-scale DNA methylation profiling methods. Bisulfite sequencing is generally accepted as the ‘gold standard’ method for detection of DNA methylation [8] providing highly accurate single nucleotide resolution. Combined with second-generation high-throughput sequencing technologies bisulfite sequencing is arguably the best approach to provide the complete methylome [9], [10], [11]. Bisulfite sequencing alone, however, does not distinguish between 5-methylcytosine (5mC) and 5-hydroxymethylcytosine (5hmC). The recently published oxidative bisulfite sequencing (oxBS-Seq) [12] and Tet-assisted bisulfite sequencing (TAB-Seq) methods [13] both enable this distinction and allow mapping of 5hmC to single nucleotide resolution. However, despite the important and welcome advance, they are both still reliant on bisulfite sequencing and hence are prohibitively costly for the larger genomes when applied genome-wide and therefore may not be the appropriate and practical method of choice for large numbers of samples.

Several methods have been developed that are more applicable on a large-scale. Driven by the need to understand the strengths and limitations of each of these methods, there have been a number of comparison papers published [14], [15], [16], [17] which have included systematic assessments of six sequencing based technologies and one array based method: whole genome bisulfite sequencing (WGBS) [18], [19], [20], reduced representation bisulfite sequencing (RRBS) [21], [22], MethylC-seq [20], methylated DNA immunoprecipitation sequencing (MeDIP-seq) [23], [24], methylated DNA capture by affinity purification (MethylCap-seq) [25], methylated DNA binding domain sequencing (MBD-seq) [26] and Infinium HumanMethylation27 BeadChip® (HumanMethylation 27K) [27]. The general conclusion from these comparisons was that although all of the techniques are capable of producing accurate data with reasonable concordance, there is no overall recommendation for any one technique and the choice of method will depend on the research question being asked, the sample numbers, cost and through-put required.

Recently Illumina have released the Infinium HumanMethylation450 BeadChip® (HumanMethylation 450K). This new array, which interrogates over 480,000 of the 28 million CpG sites in the human methylome, covers over 17-fold more CpG sites than its predecessor the HumanMethylation 27K and therefore enables a more comprehensive sampling of the methylome [28], [29], [30]. The HumanMethylation 450K array was designed to provide 96% coverage of known CpG islands (based on UCSC classifications) [31], [32], 99% of RefSeq genes [33] and many other features. Reports have been published comparing the HumanMethylation 450K with the HumanMethylation 27K [29] and whole genome bisulfite sequencing [28], [30] which have shown the data produced to be both reproducible and highly accurate. The expanded number of targets makes the HumanMethylation 450K array a potentially attractive choice when considering large sample numbers and cost.

MeDIP-seq uses immunoprecipitation to enrich for the portion of the genome containing either 5-methylcytosine (5mC) or the hydroxymethylated form, 5-hydroxymethylcytosine (5hmC) depending on the antibody used, followed by high-throughput sequencing [23], [24], [34]. MeDIP-seq provides genome-wide coverage and was used to generate the first whole-genome methylation profile of a mammalian genome [24]. It has since been successfully used to provide methylation profiles of several tissues including human breast cancer cells [35], peripheral blood mononucleocytes (PBMCs) [16] and benign and malignant nerve tumours [36].

Our study presents the first comparison of the targeted approach of the Infinium HumanMethylation450 BeadChip® with MeDIP-seq. The strengths and limitations of each method, in terms of coverage, resolution and accuracy will be explored, and regions of the methylome that can be assayed by both methods and those that are only assayed by one method determined. Discovery of differentially methylated regions by both techniques will also be examined.

## Results

We have designed a study to compare the targeted approach of the Infinium HumanMethylation450 BeadChip® with a genome-wide method, MeDIP-seq, in order to assess and compare the ability of each method to assay the methylome (Figure S1). The two methods were compared using DNA from two cell lines: GM01240 (XX) and GM01247 (XY), a sibling pair of European descent (see Methods), generating a total of four methylation profiles for analysis. Two different methods of analysis were tested per technique. On the basis of these results (data not shown), for this study, the HumanMethylation 450K data was analysed using GenomeStudio and custom-written scripts, and the MeDIP-seq data using MEDIPS (see Methods). In addition, a subset of data from each methylation profile was validated by comparison to clonal bisulfite sequencing data (used as the gold standard) from 34 CpG islands on the human X chromosome.

DNA methylation profiles were generated using the Illumina Infinium® HumanMethylation450 BeadChip® and MeDIP-seq for the two cell lines, GM01240 and GM01247 as described (see Methods). MeDIP was performed on a DNA sample from each cell line using the Methylated-DNA IP kit (Zymo Research) (see Methods). The resultant paired-end libraries were sequenced to saturation on one lane of an Illumina HiSeq 2000 using 75 cycles (see Figure S2 for saturation curve and Table S5 for sequencing statistics). This resulted in 240 M reads (18 Gb) for sample GM01240, 190 M of which were mapped with a mapping quality score of ≥10, and 234 M reads (17.6 Gb) for sample GM01247, 181 M of which mapped with a mapping quality score of ≥10. The amount of sequence data generated in our study has given an average read depth of 9.56 and 9.41 over all CpG sites in the human genome for GM01240 and GM01247 respectively. The percentage of genomic CpG sites covered by our data at different fold coverage is shown in Figure S3.

**Figure 1:**
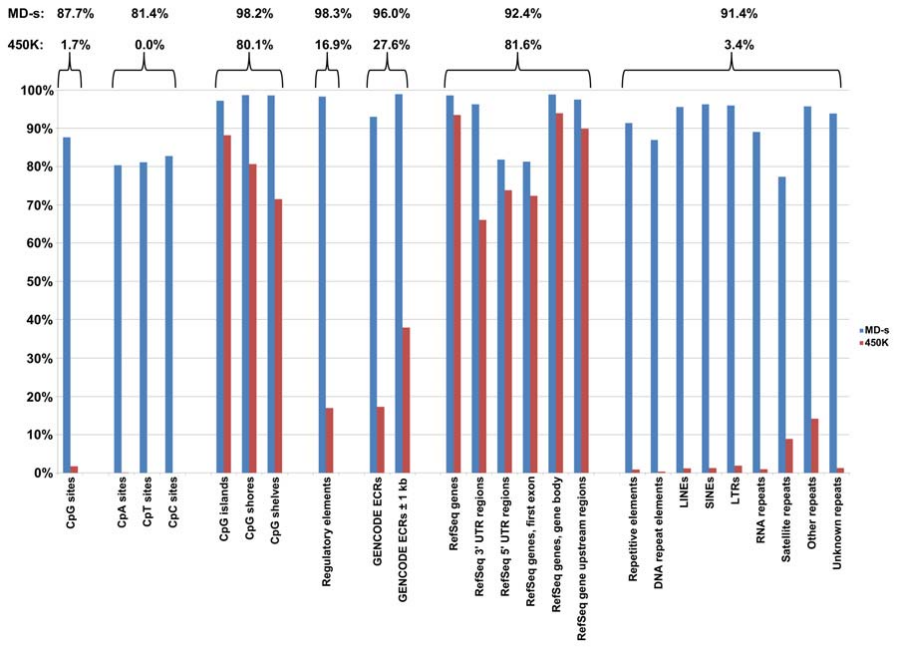
Coverage by MeDIP-seq and the HumanMethylation 450K BeadChip of different genomic features. The different features are described along the bottom axis. 100% coverage is defined as covering all of the elements of a particular type in the human genome. Coverage for MeDIP-seq data (MD-s) (averaged for GM01240 and GM01247) is shown as blue bars and for the HumanMethylation 450K (450K) as red bars. Average percentages covered for each technique for each group of features are given above the bar chart. For MeDIP-seq the region or feature was defined as being covered if any part of the region or feature was covered by or overlapped any part of one or more sequencing reads. The coverage for the MeDIP-seq was consistent between the two samples (see Table S1), illustrating a high degree of reproducibility for the technique. The coverage shown for the HumanMethylation 450K is reported as the number of features where at least one probe present on the array mapped within the features under consideration i.e. is based on the array design.

In order to evaluate how much of the genome (and methylome) is covered effectively by each method, the theoretical maximum number of sites for different features of the genome was calculated (see Figure 1 and Table S1) (see Methods for details) along with their coverage by each method. For MeDIP-seq, the region or feature was defined as being covered if any part of the region or feature was covered by, or overlapped with, one or more sequencing reads with a mapping quality score ≥10. The coverage of different genomic features by MeDIP-seq data was consistent between the two samples (see Table S1), illustrating a high degree of reproducibility for the technique. The coverage shown for the HumanMethylation 450K is based on the array design and reported as the number of regions or features with at least one probe present on the array mapping to them.

The two techniques showed different extents of coverage with respect to the calculated genomic features as expected (see Figure 1). MeDIP-seq showed a high coverage of the majority of genomic features (approaching 100% for many of them): 87.7% of CpG sites, 97% of CpG islands and 98% of the CpG island shores and shelves, 92.4% of RefSeq genes.

The HumanMethylation 450K array coverage was comparable in the targeted areas in most categories with the exception of CpG sites, GENCODE genes and regulatory elements. The HumanMethylation 450K array interrogates a total of 485, 577 CpH sites which includes 482,421 (or 1.7%) of the 28 million CpG sites genome-wide [28]. The array targets 94% of the RefSeq gene collection (based on annotation from ENSEMBL); although this collection represents a well-annotated set of sequences, it is a very conservative group and does not describe all known genes in the human genome. This results in a lower coverage of other (non-RefSeq) genes by the HumanMethylation 450K array compared to whole genome approaches.

In order to describe the genes not included in RefSeq we used the annotation from the GENCODE consortium (part of the ENCODE project) which is responsible for the accurate annotation of all evidence-based gene features in the human genome [37]. Based on the GENCODE annotation, we generated 310,060 unique expressed cluster regions (ECRs) (see Supporting Information). Approximately fifty-eight thousands of these ECRs contained additional annotation not present in RefSeq. These 58,047 ECRs contained exons from 25,582 unique ENSEMBL and HAVANA genes, of which, 7,806 (30.5%) have an official HGNC identifier (HUGO Gene Nomenclature Committee at the European Bioinformatics Institute, http://www.genenames.org/), 2,981 (11.7%) are linked to an OMIM entry (Online Mendelian Inheritance in Man, OMIM^®^, http://omim.org/), and 3,830 (15.0%) have Gene Ontology annotation [38] (see Table S2) and could include genes that prove to be disease-associated. The ECRs also contained 17,745 genes with no external annotation and as such potentially represent novel genes. The HumanMethylation 450K array targets 38% of these unique ECRs in contrast to MeDIP-seq, which covered over 96% of GENCODE ECRs.

The HumanMethylation 450K array targets 3091 CpW sites (CpA or CpT) in the human genome. MeDIP-seq targets methyl cytosine regardless of the sequence context and will target not only CpG but also all methylated CpH sites (CpA, CpT or CpC) in the genome (Figure 1 and Table S1). In our study, 80.4%, 81.1% and 82.8% of CpA, CpT and CpC dinucleotides, respectively, were covered by MeDIP-seq data, although a proportion of this coverage may be due to their proximity to methylated CpGs. Comprehensive coverage of other CpH sites may prove to be biologically important. Methylation at different CpN sites has been described in pluripotent cells [20], [39], [40], [41], although its biological significance is currently unknown.

Looking at the repetitive elements in the genome, MeDIP-seq data provided 96% coverage of transposable elements compared to less than 2% by the HumanMethylation 450K array. A major advantage of the immunoprecipitation and sequencing based methods in comparison to the hybridisation based methods is their ability to assay the methylation status of CpG dinucleotides in repeats, which is illustrated by our results. More than 45% of the human genome is derived from transposable elements and nearly half of all CpGs fall within repetitive regions of the genome [42]. Aberrant methylation in repetitive DNA was the first epigenetic alteration shown to play a role in cancer [36], [43], [44], and there is an increasing association with the methylation state of repetitive DNA and disease [36], [45], [46], which remains an area of very active research.

In order to determine the concordance in methylation levels between the two techniques, we first validated both the HumanMethylation 450K and MeDIP-seq data by comparison to bisulfite sequencing. Thirty-four CpG islands associated with 41 genes on the X chromosome had been previously selected for analysis on the basis of their chromosomal location and reported X chromosome inactivation (XCI) status (see Supporting Information) [47]. Genomic DNA from GM01240 and GM01247 was subjected to clonal bisulfite sequencing of these islands (see Methods), resulting in between 24 and 94 molecules with sequence data per CpG island. From the 34 islands, sequence data were generated for 4386 CpG sites with an average of 65 molecules per amplicon, giving a very high depth and quality of bisulfite sequence (average bisulfite conversion efficiency of 99.81%) for the validation (Table S3). The bisulfite data were analysed with MethTools [48] (see Methods) and the percentage methylation for every CpG site assayed calculated.

**Figure 2:**
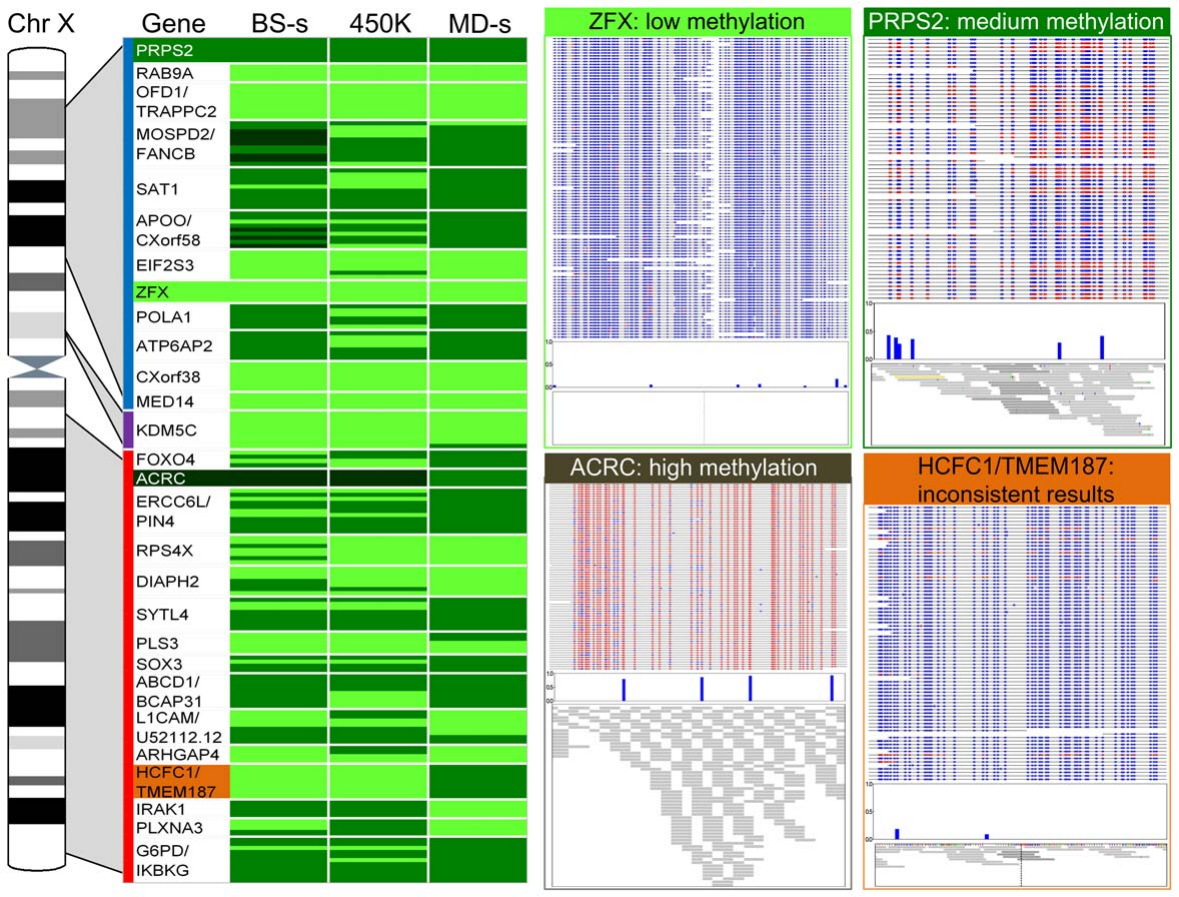
Comparison of methylation level estimates for the bisulfite sequencing, HumanMethylation 450K and MeDIP-seq data. Data are shown for the 28 islands (associated with 36 genes) containing CpG sites that overlapped with those interrogated by HumanMethylation 450K array for sample GM01240. Evolutionary strata information is shown to the right of the ideogram of the human X chromosome [66]: the blue line represents the S3 stratum; the purple line represents the S2 stratum and the red line the S1 stratum. Both names are given for genes sharing a CpG island separated by “/”. Methylation level estimates for each of the techniques are shown to the right of the gene names in light green (low), green (medium), and dark green (high). Examples of four genes are shown in more detail on the right of the figure. The gene names are highlighted in colour at the top of each panel and in a corresponding colour on the gene list. Data for the bisulfite sequencing (BS-s), HumanMethylation 450K (450K) and MeDIP-seq (MD-s) are shown at the top, center and bottom of each panel, respectively. The genes shown give examples where the three techniques agree in methylation level: low level methylation in the gene ZFX, medium level methylation in the PRPS2 gene, and a high level of methylation in the ACRC gene. Data are also given for the HCFC1/TMEM187 genes, for which different methods show inconsistency in the classified methylation levels. See Figure S4 for data for sample GM01247.

Out of the 4386 CpG sites for which sequence was generated, the positions of 326 sites were found to match exactly with CpG dinucleotides interrogated by probes on the HumanMethylation 450K array. We compared the average-beta values for each of these probes and the methylation score calculated by the MEDIPS software package [49] for the corresponding 300 bp window for the MeDIP-seq data to the methylation levels for the bisulfite data. Overall there was a strong positive correlation between both the HumanMethylation 450K and the MeDIP-seq data with the bisulfite sequencing data (Spearman correlation of 0.75 for the HumanMethylation 450K vs. bisulfite data, and 0.74 for the MeDIP-seq vs. bisulfite data, see Table 1), thus validating both methods. There was also good correlation overall of over 0.6 between the HumanMethylation 450K and MEDIPS data for these regions on the X chromosome covered by the bisulfite data. However, in some of the regions the two different methods did vary in their methylation level estimates (see HCFC1/TMEM187 in Figure 2) resulting in the lower correlation. This may be due to the fact that bisulfite sequencing and HumanMethylation 450K data both consist of methylation level estimates for single CpG dinucleotides whereas for MeDIP-seq, MEDIPS will summarise the methylation levels of all CpN sites in each 300 bp window.

**Table 1:** Concordance of the HumanMethylation 450K (450K) and MeDIP-seq (MD-s) data with bisulfite sequencing (BS-s) data.

Dataset		Concordance
Regions with bisulfite data (X chromosome)	BS-s – 450K	0.75
	BS-s – MD-s	0.74
	450K – MD-s	0.62
Whole genome data (autosomal chromosomes only)	450K – MD-s (GM01240)	0.68
	450K – MD-s (GM01247)	0.68

The top part of the table gives the concordance of the average beta-values for the 326 probes on the X chromosome from the HumanMethylation 450K (450K) and the methylation score calculated by the MEDIPS software for the MeDIP-seq data (MD-s) to the methylation levels for the bisulfite data (BS-s) from MethTools. The second half of the table contains the concordance for a similar analysis for the HumanMethylation 450K and MeDIP-seq data for all autosomal chromosomes.

Having validated the HumanMethylation 450K and MeDIP-seq data, a similar correlation analysis was done across the autosomal chromosomes to look at the genome-wide concordance between the HumanMethylation 450K array and MeDIP-seq (Table 1). There was a good positive correlation between the two methods (Spearman correlation of 0.68) for both samples indicating good agreement between the methods on a genome-wide level, supporting our observations for the X chromosome.

In order to simplify the comparison between the three different data sets we tried an alternative approach. The methylation levels for each individual CpG site assayed by the bisulfite data were classified as low, medium or high (L/M/H) based on the output from MethTools (see Supporting Information). The boundaries for low, medium and high methylation for each probe for the HumanMethylation 450K array or window for the MEDIPS data were determined by comparison to the bisulfite sequencing data (see Supporting Information). All overlapping loci on the X chromosome from the different methods were then compared (Table S4). We observed very good agreement between the different methods (see Figure 2 and Figure S4: 88% of sites were classified identically between the bisulfite sequencing data and the HumanMethylation 450K data, and 84% between bisulfite sequencing data and MeDIP-seq data (Table 2). There was 80% agreement between the HumanMethylation 450K data and MeDIP-seq data (see Table 2).

**Table 2:** Concordance of the HumanMethylation 450K (450K) and MeDIP-seq (MD-s) data with bisulfite sequencing (BS-s) data using an interval-based approach.

Dataset		Concordance
Regions with bisulfite data (X chromosome)	BS-s – 450K	0.88
	BS-s – MD-s	0.84
	450K – MD-s	0.80
Whole genome data (autosomal chromosomes only)	450K – MD-s (GM01240)	0.62
	450K – MD-s (GM01247)	0.61

The methylation levels for each individual CpG site assayed by the bisulfite data (BS-s) were classified as low, medium or high based on the output from MethTools. The boundaries for low, medium and high methylation intervals for each probe for the HumanMethylation 450K array (450K) and corresponding window for the MEDIPS data were determined by comparison to the bisulfite sequencing data. In the top part of the table the concordance between these intervals was calculated for all overlapping loci from the different methods for the regions covered by the bisulfite data. The second half of the table contains the concordance for a similar analysis for all autosomal chromosomes for the MeDIP-seq (MD-s) and HumanMethylation 450K data.

We looked at the regions that showed the highest differences between the HumanMethylation 450K and MeDIP-seq data and the clonal bisulfite sequencing data. Considering the HumanMethylation 450K data, in 40 out of 326 (12%) individual CpG sites, the methylation level was estimated to be essentially different. In 28 out of those 40 sites (70%), the estimated 450K methylation level was lower than the corresponding BS methylation level. This suggests that in semi- or highly methylated sites, the HumanMethylation 450K tends to systematically underestimate the methylation level. Furthermore, in 0.9% of those loci, the methylation level estimates were directly conflicting (Low vs. High) and in all those conflicting cases the methylation level was estimated as “High” in the bisulfite sequencing data and “Low” in the HumanMethylation 450K data.

A similar analysis was carried out for the MeDIP-seq data; in 16 out of 136 (11.8%) MEDIPS windows which overlapped with the clonal bisulfite sequencing data, the methylation level was estimated to be different. In 10 out of those 16 sites (62.5%), the estimated MEDIPS methylation level was lower than the corresponding BS-s methylation level. Furthermore, when comparing BS-s and MEDIPS there were no regions with directly conflicting (low vs. high) methylation level estimates.

We also looked at the GC content in the regions demonstrating the highest differences in methylation level estimates between the HumanMethylation 450K and MeDIP-seq. The average GC content for the MEDIPS windows was 47.5%, very similar to 49.5% for the HumanMethylation 450K data. This suggests that GC content is unlikely to be contributing to these differences.

Using the L/M/H interval-based approach we looked at the correlation between HumanMethylation 450K data and MeDIP-seq data over the autosomal chromosomes. The two methods showed a correlation above 0.6 (Table 2) with 63% of loci classified identically for GM01240 and 61% for GM01247 loci classified identically. Only 28,796 (6%) of loci in GM01240 and 16,715 (4%) of loci in GM01247 had directly conflicting (low vs. high) methylation estimates. Without additional experimental validation it is not possible to say definitively which technology is the more correct estimate of methylation levels in the regions of conflict. However, looking at those loci that are in direct conflict, and averaging over the two samples, 5.5% of loci that are estimated as “Low” by MEDIPS are estimated as “High” by the 450K array and 94.5% of loci estimated as “High” by MEDIPS are estimated “Low” by the 450K array. This suggests that either MEDIPS systematically overestimates the methylation level in regions that have no or low methylation levels or that 450K systematically underestimates methylation levels in regions that are highly methylated.

Both the HumanMethylation 450K array and MeDIP-seq have been previously shown to be capable of detecting differential methylation in the human genome [24], [29], [30], [35], [36]. The total number of differentially methylated loci detected by the HumanMethylation 450K array will be less than the number detected by MeDIP-seq because of the limitations of the targeted design versus the whole genome approach.

To determine differential methylation using the HumanMethylation 450K array, we used a similar approach to that of Sandoval *et al.*, (2011) and Dedeurwaerder *et al.*, (2011) (see Methods). Using our criteria, we found 8070 methylation variable positions (MVPs) [50] between samples GM01240 and GM01247 (excluding the Y chromosome), of which 5296 were located on autosomal chromosomes.

Differential methylation analysis was carried out on the MeDIP-seq data using the MEDIPS program (see Methods). Using our selection criteria, we detected 15,709 significant DMRs (excluding the Y chromosome) between samples GM01240 and GM01247, of which, 8244 were autosomal in origin. A large number of the autosomal DMRs (7991) overlapped partially with different repetitive elements of the genome. Nearly half of the CpGs in the human genome are known to be located in repetitive regions [42], which is supported by our results. It is thought that methylation of repetitive elements in the human genome is biologically relevant and is a possible mechanism for control of active retrotransposons [51].

Interestingly, 5873 (71%) of the autosomal DMRs detected by MEDIPS were hypermethylated in the male sample (GM01247) and 2371 (29%) were hypermethylated in the female sample (GM01240). In the HumanMethylation 450K data, 58% of MVPs were hypermethylated in the male sample and 42% in the female sample. These data support the idea that there is a wealth of non-sex chromosome genes that are differentially methylated (and therefore potentially also differentially regulated) between males and females [52].

**Figure 3:**
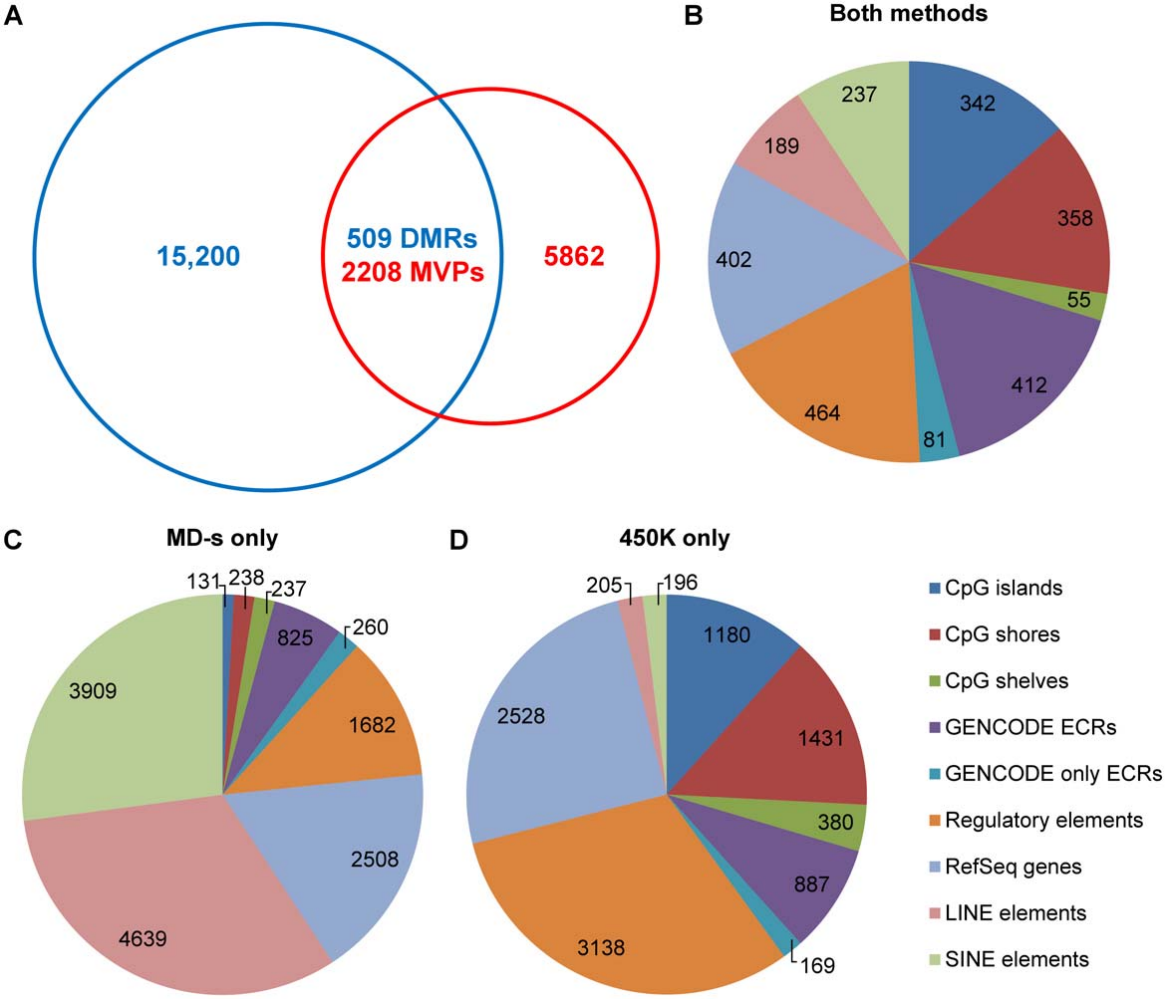
Differentially methylated loci detected by the HumanMethylation 450K array (450K) and MeDIP-seq (MD-s). (A) The numbers of methylation variable positions (MVPs) detected the by the 450K array (autosomal and X) are given in the red circle, and the number of differentially methylated regions (DMRs) detected with MD-s in the blue circle. The number of differentially methylated loci detected by both methods is given in the intersection of the two circles. Any DMR detected by MD-s within this intersection contained one or more significant MVP(s) detected by the 450K array. (B) Location with respect to different genomic features of the differentially methylated regions detected by both methods (see key to the bottom right of the figure for the genomic feature represented by each colour). (C) Exact number of and location with respect to different genomic features of the DMRs detected by MD-s only. (D) Exact number of and location with respect to different genomic features of the MVPs detected by 450K only. It is important to note that many of the genomic elements overlap with each other. Additionally, in the case of the DMRs found by MD-s, many of the longer DMRs span several different genomic features.

To look at the concordance of the differential methylation detected by the two methods, we compared the MVPs found with HumanMethylation 450K array to the DMRs found with the MeDIP-seq data (see Supporting Information). Out of the total of 8070 MVPs detected with the HumanMethylation 450K platform, 2208 overlapped with 509 significant DMRs found with MeDIP-seq (Figure 3A). The overall concordance for the overlapping MVPs/DMRs was good: in 97.4% of differentially methylated loci found by both methods the direction of the differential methylation agreed. Figure 3B shows the location with respect to different genomic features of the DMRs detected by both techniques.

5203 autosomal only MVPs were detected by the HumanMethylation 450K array only, i.e. none of them overlapped with a significant DMR found using MEDIPS. On inspection, 4911 (94%) of these were covered by MeDIP-seq reads and had been detected by the MEDIPS DMR analysis but had been excluded as lower confidence DMRs. Conversely, 8181 autosomal DMRs were detected by MeDIP-seq only. Of these, 1030 (12.6%), had one or more HumanMethylation 450K probes that overlapped with them but had been excluded as being not significant MVPs. Figures 3C and 3D illustrate the genomic features covered by the differentially methylated loci detected by MeDIP-seq or the HumanMethylation 450K respectively. The exact number of DMRs or MVPs found in different genomic features is also given. It is important to note that many of the genomic elements overlap with each other. For example, for MD-s, the number of significant DMRs that overlap with LINE elements is 4639 as shown. However, the number of DMRs that overlap with LINE elements which do not overlap with any other genomic feature is 2767. Additionally, in the case of the DMRs found by MD-s, many of the longer DMRs span several different genomic features.

Our results show that for methylation level determination both the Infinium HumanMethylation450 BeadChip® and MeDIP-seq are reliable and correlate well with each other. Both methods can be used successfully to determine differentially methylated loci in RefSeq genes, CpG islands, shores and shelves. MeDIP-seq however interrogates more regions of the human genome, including non-RefSeq genes and repetitive elements, allowing the detection of nearly twice as many differentially methylated regions.

## Discussion

One of the important driving forces in the study of epigenetics is the impact of epigenetics in disease. There are still relatively few common diseases that have been explained fully by conventional single nucleotide polymorphism (SNP), indel (short insertions/deletions) and copy-number variant (CNV) analyses [5]. Some of the remainder could potentially be explained by alterations in DNA methylation patterns. It has also been suggested that environmental influences on epigenetic modifications could be an important factor in disease risk [3].

The study of epigenetics is expanding rapidly with several large-scale projects underway analysing large collections of samples from different tissues (IHEC: http://www.ihec-epigenomes.org/index.html; NIH Roadmap Epigenomics Mapping Consortium: http://www.roadmapepigenomics.org; BLUEPRINT, http://www.blueprint-epigenome.eu). Growth in this field has driven technological developments to increase the capacity and efficiency of the methods available for studying the methylome. Selection of the appropriate method is imperative and, with increasing importance, should enable comparison of data produced by the large international collaborative efforts currently underway. With this in mind, our study presents a comparison between the targeted approach of the Infinium HumanMethylation450 BeadChip® and MeDIP-seq, an immunoprecipitation and sequencing- based method covering the whole genome.

Our results show that both methods are capable of providing accurate and robust results. One of the fundamental differences between the two is the coverage of the genome and methylome provided. MeDIP-seq can detect methylation at any methylated CpN site in the genome and is therefore theoretically capable of 100% coverage of the methylome. Along with other whole genome-based techniques, this makes MeDIP-seq very useful for hypothesis free discovery of methylation states and changes therein. As with other sequencing based techniques, there are some limitations due to the challenge of mapping sequencing reads accurately within highly repetitive and complex regions of the genome (and hence the methylome). Nevertheless, use of MeDIP-seq and other genome-wide methods will inevitably lead to a more complete picture of the methylome than the targeted approach. In addition, the ability to detect regions of non-CpG differential methylation may be biologically relevant for the assessment of the effect of methylation in pluripotent cell types [41]. There are a significant number of genes and other genomic features that are not targeted by the HumanMethylation 450K array that are potentially of functional significance. Hence the HumanMethylation 450K is not as suitable for hypothesis free discovery or for detecting methylation outside of RefSeq genes and other known features.

A major advantage of sequencing-based methods such as MeDIP-seq is the ability to interrogate repetitive elements (nearly 50% of the CpGs in the genome fall in repetitive regions). These CpGs are not easily assayed by array-based methods due to the problems of cross-hybridisation, a limitation of array-based techniques and will have implications for the study of some diseases where methylation in these regions is an important factor.

MeDIP-seq is a relatively low resolution technique that can detect methylated regions of approximately 150–200 bp rather than the individual single nucleotide sites detected by the HumanMethylation 450K. Although the highest possible resolution of a single base-pair is desirable, the methylation state of neighbouring CpG sites has been shown to be highly correlated over distances as great as 1000 bp [53], [54]. Depending on the research question it may not be absolutely necessary to have single base pair resolution and the resolution provided by MeDIP-seq may be sufficient.

The analysis of methylation data requires an understanding of the differences in resolution between methods used. MeDIP-seq provides regional methylation information, likely to arise from several MVPs. In the HumanMethylation 450K array, single CpG sites are assayed providing exact positional information. These sites are likely to have methylation levels indicative of the regional methylation surrounding the MVP given that methylation levels have been shown to be correlated within regions of up to 1 kb. It is possible to perform a more regional analysis using single site based array data using custom arrays designed to cover multiple consecutive clustered MVPs. This approach, CHARM, (Comprehensive High-throughput Arrays for Relative Methylation) [55] was developed to allow genome weighted smoothing and more confident calling of DMR from array data [55]. Application of the CHARM method may be possible with future higher density designs of the Infinium array enabling clustering of MVPs. CHARM arrays have recently been technologically extended to encompass significantly larger numbers of CpGs up to 5.2 million CpG sites [56] and although this would not cover all 28 million CpGs in the human genome, this method may offer a suitable alternative that has some of the benefits of MeDIP-seq with the flexibility and cost efficiency of an array.

Our results also show that both techniques are capable of detecting differential methylation. As expected, the number of differentially methylated autosomal loci detected by the targeted method (HumanMethylation 450K) was smaller than with the hypothesis-free approach of MeDIP-seq (5296 vs. 8244 respectively). It is important to note that the HumanMethylation 450K array will detect differentially methylated sites (not regions) between compared samples. Conversely, with MeDIP-seq it is only possible to detect differentially methylated regions and not possible to detect single differentially methylated sites, requiring additional analysis to determine the state of the individual CpN sites involved.

The HumanMethylation 450K has been designed to facilitate the processing of large numbers of samples in a high-throughput and cost-effective manner. MeDIP-seq is commonly thought to be more costly and less amenable to large sample numbers but can be successfully automated [57]. Taiwo et al., reported that 60 million high-quality reads would cover up to 70% of all CpGs at a minimum of 1× and 30% of all CpGs at a minimum of 10× [58]. Typically 60–80% of CpGs in the genome are methylated. With this is mind Taiwo et al., suggest that a typical MeDIP-seq experiment would interrogate most methylated CpGs at 1× [58]. Li et al., (2010) estimated that 3 Gb of data are sufficient to study the methylome using MeDIP-seq [16]. This estimate is in broad agreement with the number of reads required as suggested by Taiwo et al., [58]. The amount of sequence data generated in our study is therefore more than the recommended minimum amount required to give good coverage of the methylome. Based on these figures MeDIP-seq samples could be indexed allowing multiplexing of up to 6 samples per Illumina HiSeq lane at the current level of data production. This has a positive effect on cost such that MeDIP-seq becomes at least 18× more cost effective per CpG dinucleotide than the HumanMethylation 450K. As a consequence sequencing-based enrichment methods should not necessarily be thought to be prohibitively expensive for looking at the methylome.

Looking at our results, the dynamic range of HumanMethylation 450K and MeDIP-seq data appears to be less than that of bisulfite sequencing. For MeDIP-seq, this is partially due to the software used to analyse the data, MEDIPS. In any one window, MEDIPS will average out or “smooth” peaks of methylation resulting in a smaller dynamic range. This may give the false impression that MeDIP-seq is less efficient at capturing highly methylated regions of the genome. However, this is an artefact of the data analysis. Analysis of MeDIP-seq data remains problematic despite there being several methods available [24], [59], [60].

The evolution and development of second-generation sequencing has revolutionised how we study DNA methylation. The once impractical, costly whole genome experiment can now be performed in a more cost- and time- efficient manner using methods such as MeDIP-seq (and others) that reduce the amount of sequencing needed and increase the coverage of data generated for methylated regions of the genome. For small numbers of samples or smaller genomes, it is possible to sequence an entire bisulfite-converted genome [18], [19], [20], [54]. However, despite the reduction in costs and increase in sequencing yields, price and throughput still remains a barrier to researchers wishing to carry out large-scale studies with a sufficient depth of coverage. The array-based HumanMethylation 450K provides an accessible alternative capable of producing data from large numbers of samples and allowing cross sample comparisons relatively easily but is limited by its targeted design. Although the Infinium HumanMethylation450 BeadChip® is an improvement on its predecessor (the Infinium HumanMethylation27 Bead-Chip®), our study has shown that it may not be the most comprehensive tool for studying the methylome but will deliver affordable robust and standardised data in those regions that it targets allowing easier comparison between different studies. Additionally it is relatively simple to integrate the HumanMethylation 450K data with genotyping data from genome-wide association studies and gene expression data to look at the relationship between genotype, methylation and expression. However, analysis of HumanMethylation 450K data has proved to be challenging with significant issues around QC and data normalisation. New methods for analysis are continually being developed and published, each of which has its merits. Whilst great progress has been made it is still not clear which of the available methods is the most suitable [61].

In summary, MeDIP-seq would be the method of choice when the impact of, and/or genomic location of, methylation in the samples under study is unknown or is likely to be outside of RefSeq genes, for example in repetitive regions of the genome. MeDIP-seq is automatable and as sequencing costs continue to fall, and DNA requirements for MeDIP-seq decrease, it is becoming more suitable for larger sample sets and a wider range of samples including precious clinical samples where DNA may be limited. It is also capable of analysing both 5mC and 5hmC in contrast to the HumanMethylation 450K. The HumanMethylation 450K BeadChip® is currently more amenable for high-throughput experiments with large number of samples. It would be a suitable method of choice if the methylation or change of methylation state in question is thought to be in well-characterised genes. It would also be the method of choice when comparing results across studies and integrating methylome data with, for example, array-based expression data. The coverage of RefSeq genes is very good for the HumanMethylation 450K BeadChip®, however, coverage of other regions of the genome is more limited, and, limited to the study of 5mC.

Currently no single method provides what is ultimately required for the study of the methylome – a cost-effective, unbiased method requiring a small amount of input sample and which will allow processing of large numbers of samples and detect (and distinguish between) all methylation marks including 5mC and 5hmC at single nucleotide resolution in one experiment. Whilst we are moving towards being able to detect DNA methylation and other DNA modifications directly using nanopores [62] and single-molecule, real-time sequencing methods [63], [64] there are still significant challenges to address before these methods are a practical choice for high throughput methylation projects. We will therefore continue to need to make choices about the best available methods for our investigations whilst considering the scope and detail of the biology we aim to unravel.

## Methods

### Ethics statement

This study has been approved by the ethics committee of the Wellcome Trust Sanger Institute (WTSI). This study only used extracted DNA from cell-lines, which falls outside of the UK Human Tissue Act.

### Genomic DNA samples and DNA extraction

The two cell lines used in this study, GM01240 (XX) and GM01247 (XY), (a sibling pair of European descent), were sourced from the NIGMS Human Genetic Cell Repository at the Coriell Institute for Medical Research. Genomic DNA (gDNA) was extracted from cell pellets using a DNeasy Blood and Tissue kit (Qiagen) according to the manufacturer's protocol including treatment with RNaseA and Proteinase K.

### Calculation of number of sites of different features in the genome

The coordinates for CpG islands, regulatory elements, RefSeq genes (and other related elements) and human repetitive elements were downloaded from the ENSEMBL database (v63). CpG island shores were calculated at 2 kb either side of an island and CpG shelves as the 2 kb extending from the shores. The number of CpG shelves is fewer than the number of CpG shores because if two CpG islands are less than 4 kb apart they will be separated by one or two shores but no shelves. Coordinates for GENCODE ECRs were calculated from the GENCODE database (v8) by collapsing all overlapping GENCODE genes into expressed cluster regions (see Supporting Information). Coordinates for all CpN sites were extracted from the GRCh37 1000 Genomes reference genome (ftp://ftp.1000genomes.ebi.ac.uk/vol1/ftp/technical/reference/human_g1k_v37.fasta.gz).

### Methylated DNA immunoprecipitation sequencing (MeDIP-seq)

Five micrograms of gDNA were randomly sheared to a median fragment size of 200 bp using a Covaris S220. Illumina Paired End libraries were prepared as described in the manufacturer's protocol except for the replacement of the gel purification and size selection step in the final stage with purification using Agencourt AMPure XP SPRI beads (according to the supplier's protocol). The resulting libraries were assessed for quantity and size using an Agilent 2100 Bioanalyser and DNA 1000 assay.

Methylated DNA immunoprecipitation was performed using the Methylated-DNA IP kit (Zymo Research) using a 1∶10 ratio of DNA to antibody. Before precipitation 480 ng of the Illumina Paired End library (Input DNA) were diluted in DNA denaturing buffer to a final volume of 60 µl and denatured at 98°C for 5 minutes. 5 µl (40 ng) were retained for use as an input control and purified using a MinElute PCR Purification Kit (Qiagen) with a final elution into 15 µl elution buffer (EB). 50 µl (400 ng) of denatured sample were used in the immunoprecipitation (IP) reaction which was incubated for 1 hr at 37°C @ 700 rpm, purified according to the manufacturers' instructions and eluted in 15 µl DNA elution buffer. MeDIP efficiency was assessed by performing comparative Ct quantitative PCR using the IP and input samples as template, Power SYBR green PCR master mix (Applied Biosystems) and primers supplied by Diagenode (see Supporting Information for primer details). qPCR was performed using a StepOnePlus Real-Time PCR system (Applied Biosystems) with the following cycling conditions: 95°C for 10 mins, followed by 40 cycles of 95°C for 15 sec; 60°C for 1 min and concluded with melt curve analysis. MeDIP efficiency was calculated using the manufacturer's formula (see Supporting Information for details). MeDIP percentage recovery and percentage specificity results for GM01240 and GM01247 can be seen in Table S6.

A library enrichment step was performed using 10 cycles of PCR according to the Illumina Paired End Sample Prep protocol and the resulting libraries assessed for quantity and size using the Agilent 2100 Bioanalyser and DNA 1000 assay. Each MeDIP-seq library was sequenced as paired-end to saturation on one lane of an Illumina HiSeq 2000 using 75 cycles (see Figure S2). The raw sequencing reads are available through the European Genome-Phenome Archive (http://www.ebi.ac.uk/ega, accession to be supplied).

Sequence reads were aligned to the human reference genome (1000 Genomes version of GRCh37) using Burrows–Wheeler alignment (BWA). Duplicates were mapped with Picard MarkDuplicate tool (see Table S5 for sequencing statistics for each sample). High quality sequence data (reads with a mapping quality score of ≥10) were analysed using the MEDIPS software package [49] to estimate methylation levels. Estimates were calculated for all consecutive 300 bp windows across the genome for each sample. Differential methylation analysis was carried out using MEDIPS in 500 bp windows excluding regions with no read coverage in both samples. Genomic regions with at least three consecutive windows that were statistically significantly differentially methylated between compared samples were considered to be differentially methylated regions (DMRs).

### Genome-wide methylation profiling using the Illumina Infinium HumanMethylation450 BeadChip

Bisulfite conversion of 1 µg of the DNA sample was performed using the EZ-96 DNA Methylation Kit (Zymo Research) according to the manufacturer's protocol. 5 µl of the bisulfite converted DNA were used for genome-wide methylation profiling using the Illumina Infinium HumanMethylation450 BeadChip according to manufacturer's protocol. Post-hybridisation and washing, the BeadChips were scanned using the Illumina HiScan SQ scanner. The efficiency of the bisulfite conversion was checked according to the manufacturer's protocol.

Raw image data were imported into the GenomeStudio v2010.3 Methylation module (version 1.8.2. using the human genome reference version 37) which was used to extract the fluorescent signal intensities and normalise the data. Briefly, background signal intensity was subtracted from the raw signal intensity values and data were normalised with respect to the Illumina internal control probes. The methylation level for each probe was calculated as an average beta-value scaled from 0 to 1 (*AVG_Beta*) representing the ratio of the intensity of the methylated beads to the combined locus intensity. Results of this analysis were exported from GenomeStudio as standard report files and further analysed with custom scripts.

To detect methylation variable positions (MVPs) we used a similar approach to that described in Sandoval *et al.*, (2011) and Deduerwaerder *et al.*, (2011). Methylation levels, estimated as average beta values, for both samples, were compared in a probe-wise manner and any locus for which the corresponding methylation level estimates (*AVG_Beta*) differed by at least 2-fold and by 0.2 or more was considered to be differentially methylated. We added the additional requirement of an absolute difference of 0.2 in *AVG_Beta* values to avoid the situation where CpG sites with methylation level estimates of 0.01 in one sample and 0.02 in the other sample would be called as differentially methylated despite the ≥2-fold difference criteria being fulfilled.

### Clonal bisulfite sequencing

Genomic DNA from GM01247 and GM01240 was prepared for bisulfite treatment by fragmentation by restriction digestion. Digests were performed using 18–25 µg DNA with 100 U Hind III in 1× NEB2 buffer and incubated at 37°C overnight. Digested DNA was purified using phenol: chloroform: isoamyl alcohol extraction followed by an ethanol/sodium acetate precipitation step. 1.2 µg of digested DNA were subjected to bisulfite conversion using the EZ DNA Methylation Kit™ (Zymo Research). 25–50 ng of bisulfite converted genomic DNA were used as a template for Hot-start PCR using primers designed to amplify selectively the bisulfite converted DNA across targeted CpG islands (see Table S3 for primer sequences and Supporting Information for details of primer design and validation and amplification conditions).

PCR products were purified prior to cloning using a MinElute PCR Purification Kit (Qiagen), and then ligated into the pGEM®-T Easy Vector (Promega). Transformations were performed using One Shot® Mach1™ T1 Phage-Resistant Chemically Competent *E. coli* (Invitrogen). Cells were plated onto selective agar containing Ampicillin and X-gal and incubated at 37°C overnight. A small number of colonies were checked by colony PCR using standard protocols. 96 colonies from each ligation (amplicon) were subjected to an automated 96-well plasmid prep using standard protocols. The resulting DNA was sequenced using M13 forward and reverse primers and ABI Prism Big Dye Terminator v3.1 chemistry. Reactions were run on ABI 3730 sequencers with the ABI base-calling algorithm KB Basecaller.

Sequence data were assembled into a GAP database [65] (version 4), and manually curated to remove poor quality data from the assembly. For each amplicon the remaining high quality data were exported in FASTA format and aligned to *in silico* bisulfite converted reference sequence and unconverted genomic sequence using the ClustalW 1.8 (http://searchlauncher.bcm.tmc.edu/multi-align/Options/clustalw.html). Text files were generated containing the aligned and original parent sequences from each amplicon and submitted to MethTools to calculate the percentage of methylated molecules for each CpG assayed [48] (http://genome.imb-jena.de/methtools/).

## Supporting Information

Figure S1 Outline of the comparison of MeDIP-seq to the HumanMethylation 450K array. The two methods were compared on DNA from two cell lines; GM01240 (XX) and GM01247 (XY), a sibling pair of European descent (see Methods), generating a total of four methylation profiles for analysis. Two different methods of analysis were tested per technique: For the HumanMethylation 450K we tested both GenomeStudio in combination with custom-written scripts as well as the IMA package [67]. For the analysis of the MeDIP-seq data we tested both MEDIPS and Batman. In addition, a subset of data from each methylation profile was validated by comparison to clonal bisulfite sequencing data from 34 CpG islands on the human X chromosome which was used as the gold standard.
(TIF)

Figure S2 Saturation analysis of MeDIP-seq data for samples GM01240 (A) and GM01247 (B). Results from the MeDIP-seq satuaration analysis calculated by part of the MEDIPS package that checks if the number of input regions (MeDIP-seq sequencing reads) is sufficient to generate a saturated and reproducible methylation profile for the analysed sample(s). Note that for both samples, lines depicting actual saturation and estimated saturation overlap to a great extent.
(TIF)

Figure S3 Sequencing depth and CpG coverage. The percentage of genomic CpG sites covered at different depths of sequence (fold coverage) is shown for GM01240 (240 M reads, 18 Gb of sequence ) in dark blue; for GM01247 (234 M reads, 17.6 Gb ) in green; for GM01240 (120 M reads, 9 Gb ) in blue; for GM01240 ( 60 M reads, 4.5 Gb) in light blue; for Sample#1 (82 M reads, 6.3 Gb ) in yellow; for Sample#2 ( 74 M reads, 5.7 Gb ) in red. It is important to note that Sample#1 and Sample#2 (unrelated to this study but processed in the same way) were both sequenced on one lane of an Illumina GAII resulting in a lower sequencing yield compared to GM01240 and GM01247 which were both sequenced on one lane of an Illumina HiSeq 2000. The data for the 120 M and 60 M reads graphs for GM01240 were calculated from the 240 M dataset by taking a subset of the reads equivalent to half (1/2) and a quarter (1/4) of the original dataset. All experimental datasets were sequenced to saturation according to the MEDIPS saturation analysis (data for GM01240 and GM01247 are shown on Figure S2).
(TIF)

Figure S4 Comparison of methylation level estimates for the bisulfite sequencing (BS-s), HumanMethylation 450K (450K) and MeDIP-seq (MD-s) data. Data are shown for the 28 islands (associated with 36 genes) containing CpG sites that overlapped with those interrogated by HumanMethylation 450K array for sample GM01247. Evolutionary strata information is shown to the right of the ideogram of the human X chromosome [66]: the blue line represents the S3 stratum; the purple line represents the S2 stratum and the red line the S1 stratum. Both names are given for genes sharing a CpG island separated by “/”. Methylation level estimates for each of the techniques are shown to the right of the gene names in light green (low), green (medium), and dark green (high).
(TIF)

Table S1 Coverage by MeDIP-seq and the HumanMethylation 450K BeadChip of different genomic features. The theoretical maximum number of sites for different features of the genome (Sites or features in the genome) was calculated as follows: The coordinates for CpG islands, regulatory elements, RefSeq genes (and other related elements) and human repetitive elements were downloaded from the ENSEMBL database (v63). CpG island shores were calculated at 2 kb either side of an island and CpG shelves as the 2 kb extending from the shores. The number of CpG shelves is fewer than the number of CpG shores because if two CpG islands are less than 4 kb apart they will be separated by one or two shores but no shelves. Coordinates for GENCODE ECRs were calculated from the GENCODE database (v8) by collapsing all overlapping GENCODE genes into expressed cluster regions (see Supporting Information). Coordinates for all CpN sites were extracted from the GRCh37 1000 Genomes reference genome (ftp://ftp.1000genomes.ebi.ac.uk/vol1/ftp/technical/reference/human_g1k_v37.fasta.gz). The coverage shown for the HumanMethylation 450K (Sites covered by design) is based on the array design and reported as the number of regions or features with at least one probe present on the array mapping to them. The following column gives the percentage of the theoretical maximum number of sites covered for each feature by the HumanMethylation 450K. For MeDIP-seq, the region or feature was defined as being covered if any part of the region or feature was covered by or overlapped with one or more sequencing reads with a mapping quality score ≥10. The number of sites covered for each sample (GM01240 (XX) or GM01247 (XY)) is given (Sites covered by ≥1 reads) as well as the percentage of the theoretical maximum number of sites covered for each feature (Percentage of genomic sites covered).
(DOCX)

Table S2 Unique ENSEMBL and HAVANA genes with associated annotation. Based on GENCODE annotation 310,060 unique expressed cluster regions (ECRs) were generated (see Supporting Information). Approximately fifty-eight thousands of these ECRs contained additional annotation not present in RefSeq. These 58,047 ECRs contained exons from 25,582 unique ENSEMBL and HAVANA genes (column A for the ENSEMBL ID). Any external ID is given in column B. 7,806 of the ECRs (30.5%) have an official HGNC identifier (HUGO Gene Nomenclature Committee at the European Bioinformatics Institute, http://www.genenames.org/) (column C), 2,981 (11.7%) are linked to an OMIM entry (Online Mendelian Inheritance in Man, OMIM^®^, http://omim.org/) (column D), and 3,830 (15.0%) have Gene Ontology (GO) annotation (column E). The ECRs also contained 17,745 genes with no external annotation and as such potentially represent novel genes.
(XLS)

Table S3 Summary of clonal bisulfite sequencing data from 81 data sets encompassing 34 CpG islands on the human X chromosome. This table summarises the clonal bisulfite sequencing data for the 81 data sets amplifying the 34 CpG islands selected on the human X chromosome for analysis. For each amplicon the table gives the evolutionary stratum within which the CpG island being assayed lies, the official HGNC unique gene symbol for the gene or genes associated with the island and a description of the gene itself and its reported X chromosome inactivation status as described in reference 45. Data are given for the length of the CpG island in bp. Amplicon length, coordinates and the sequences of the primers used to amplify each amplicon are given. Counts are given for the number of CpG dinucleotides and CpH (CpA/CpT/CpC) dinucleotides in each amplicon. The number of sequencing reads analysed per amplicon is recorded alongwith the number of individual molecules analysed. The average number of molecules analysed per region is given as 65. The total number of cytosines analysed within the context of a CpG dinucleotide is calculated (the number of CpGs in the amplicon×the number of molecules analysed). The total number of bisulfite converted cytosines (in the context of either CpA, CpT or CpC) and the number of unconverted cytosines are given. The percentage of unconverted cytosines (in a CpA, CpT or CpC context) is calculated. The bisulfite efficiency is expressed as a percentage. The average bisulfite conversion efficiency across all 81 data sets is 99.81%.
(XLS)

Table S4 Comparison of methylation levels between bisulfite sequencing, HumanMethylation 450K and MeDIP-seq data for GM01240 and GM01247 using an interval-based approach. The HGNC unique gene symbol for the gene associated with the CpG island being assayed is given. Two gene names separated by “/” indicate two genes share the island. “XY only” indicates that there are data for sample GM01247 only. “XX” only indicates that there are data for sample GM01240 only. The coordinates on the X chromosome (GRCh37) for each individual CpG analysed by bisulfite sequencing with the percentage methylation determined by MethTools analysis of the bisulfite sequencing data are given. For the bisulfite sequencing, the methylation levels for each individual CpG site assayed were classified as low, medium or high based on the output from MethTools with low, medium or high defined as: low (L), methylation level less than 20%; medium (M), methylation level greater than or equal to 20% and less than 70%) and high (H), methylation level greater than or equal to 70%. The bisulfite sequencing data were considered to be the gold standard. The exact values used for the boundaries for the HumanMethylation 450K and MEDIPs data were found iteratively by treating the bisulfite sequencing estimated methylation level as the “true” methylation level and comparing all possible combinations of boundaries programmatically to maximise the number of correctly classified overlapping CpG sites and minimise the number of incorrectly classified overlapping CpG sites between the two datasets. For the HumanMethylation 450K methylation levels were defined as low (L), average-beta value of less than 0.21; medium (M), average-beta value of greater than or equal to 0.21 and less than 0.57, and high (H), average-beta value of greater than or equal to 0.57. For MEDIPS, methylation levels were classified as low, medium or high defined as low (L), MEDIPS score of less than 280; medium (M), MEDIPS score of greater than or equal to 280 and less than 410, and high (H), MEDIPS score of greater than or equal to 410The coordinate on the X chromosome (GRCh37) and name (supplied by Illumina) of the individual probe from the HumanMethylation450 array used in the analysis are given. The average beta-value of each probe (scaled from 0 to 1) as calculated by Genome Studio represents the methylation level for each probe from the HumanMethylation 450K. The coordinates on the X chromosome (GRCh37) of the window of MeDIP-seq data analysed by MEDIPS are given, with the MEDIPS score as a measure of the methylation level. The coordinate on the X chromosome (GRCh37) and name (supplied by Illumina) of the individual probe from the HumanMethylation450 array used in the analysis are given. The average beta-value of each probe (scaled from 0 to 1) as calculated by Genome Studio represents the methylation level for each probe from the HumanMethylation 450K. The coordinates on the X chromosome (GRCh37) of the window of MeDIP-seq data analysed by MEDIPS are given, with the MEDIPS score as a measure of the methylation level.
(XLSX)

Table S5 Sequencing statistics for MeDIP-seq data for GM01240 and GM01247. A summary of sequencing statistics for MeDIP-seq data for GM01240 and GM01247. Each MeDIP-seq library was sequenced as paired-end to saturation on one lane of an Illumina HiSeq 2000 using 75 cycles. The total number of reads post image analysis and QC are given generated for each sample followed by the number of passed reads are given. The percentage of GC found in the reads is reported. The total number of bases sequenced and the percentages of high quality bases are calculated. Sequence reads were aligned to the human reference genome (1000 Genomes version of GRCh37) using Burrows–Wheeler alignment (BWA). The raw number of mapped reads is given as well as the number of reads mapping to the reference genome. Duplicates were mapped with Picard MarkDuplicate tool.
(XLSX)

Table S6 MeDIP percentage recovery and percentage specificity for GM01240 and GM01247 based on Comparative Ct qPCR data. The efficiency of the immunoprecipitation in the MeDIP was assessed by performing comparative Ct quantitative PCR using the IP and input samples as template. The primer pairs used for the qPCR for the human TSH2B and GAPDH control genes were supplied by Diagenode. The human TSH2B gene is known to be highly methylated in all somatic cells. The human GAPDH primers span the human GAPDH promoter sequence which is known to be unmethylated. The genomic coordinates of the regions used in the analysis based in NCBI build 36 are given. The percentage recovery was calculated using the following formula from Diagenode: % (meDNA-IP/Total Input): 2∧[(Ct (10%input)-3.32) -Ct(meDNA-IP)]×100%. The percentage specificity was calculated as: 1- (enrich unmeth/enrich meth)×100%.
(XLS)
