# Supplementary figures and images for: A Comparison of the Whole Genome Approach of MeDIP-Seq to the Targeted Approach of the Infinium HumanMethylation450 BeadChip® for Methylome Profiling
Source: PLoS One. 2012 Nov 29;7(11):e50233. doi: 10.1371/journal.pone.0050233 (PMC3510246; doi:10.1371/journal.pone.0050233)

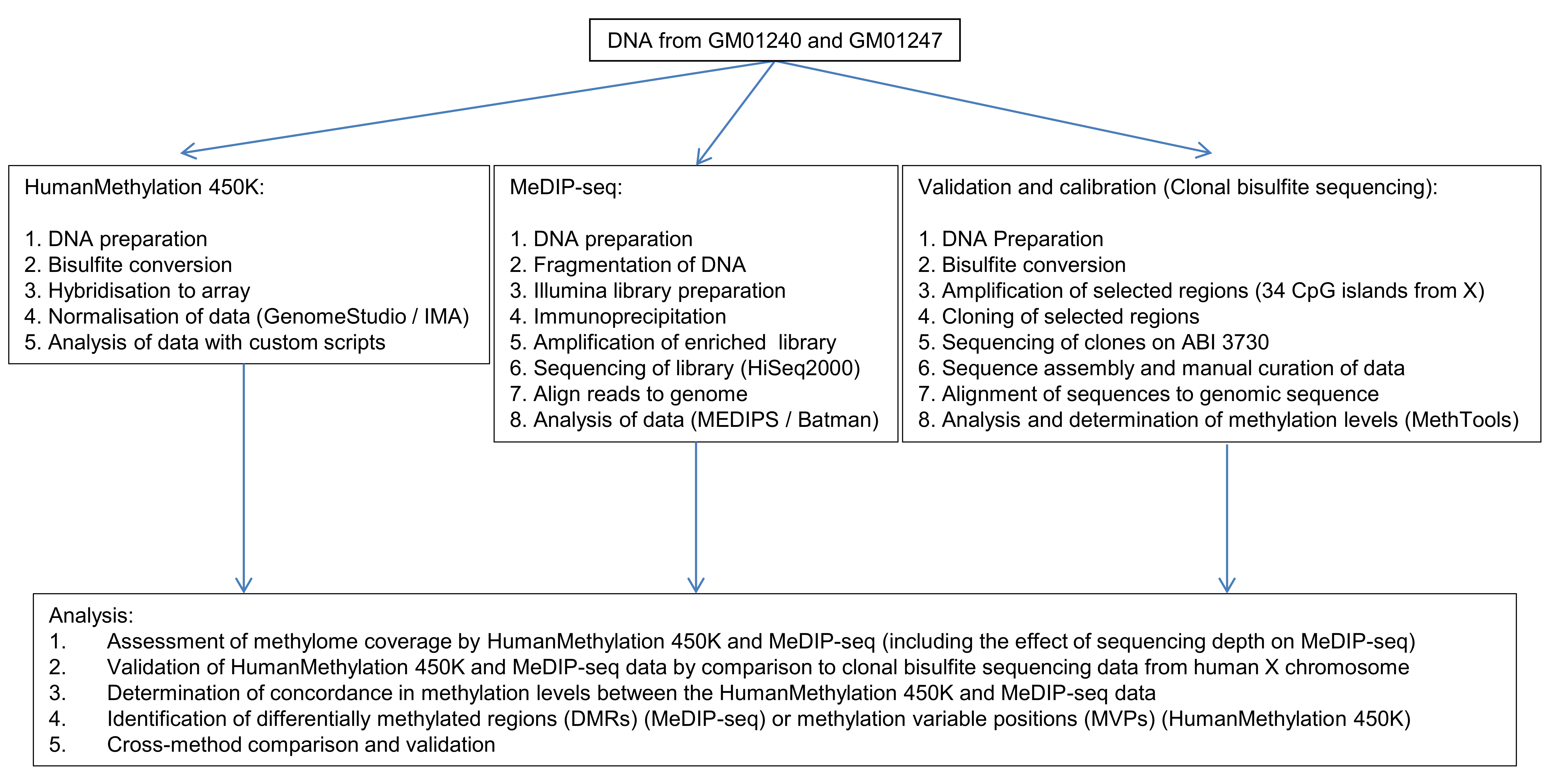

Supplement: Figure S1 — Outline of the comparison of MeDIP-seq to the HumanMethylation 450K array. The two methods were compared on DNA from two cell lines; GM01240 (XX) and GM01247 (XY), a sibling pair of European descent (see Methods), generating a total of four methylation profiles for analysis. Two different methods of analysis were tested per technique: For the HumanMethylation 450K we tested both GenomeStudio in combination with custom-written scripts as well as the IMA package [67]. For the analysis of the MeDIP-seq data we tested both MEDIPS and Batman. In addition, a subset of data from each methylation profile was validated by comparison to clonal bisulfite sequencing data from 34 CpG islands on the human X chromosome which was used as the gold standard. (TIF) [file pone.0050233.s001.tif]

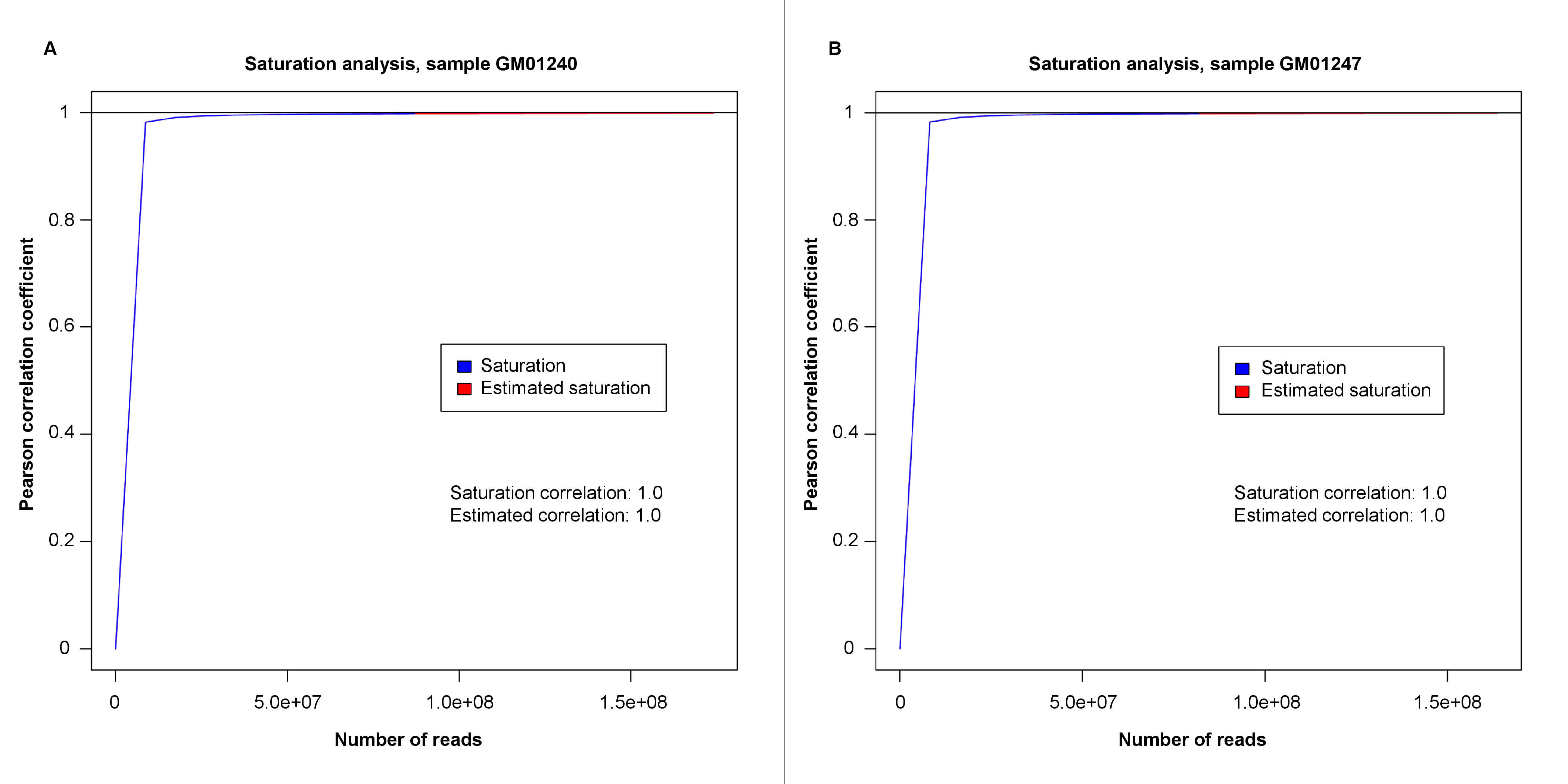

Supplement: Figure S2 — Saturation analysis of MeDIP-seq data for samples GM01240 (A) and GM01247 (B). Results from the MeDIP-seq satuaration analysis calculated by part of the MEDIPS package that checks if the number of input regions (MeDIP-seq sequencing reads) is sufficient to generate a saturated and reproducible methylation profile for the analysed sample(s). Note that for both samples, lines depicting actual saturation and estimated saturation overlap to a great extent. (TIF) [file pone.0050233.s002.tif]

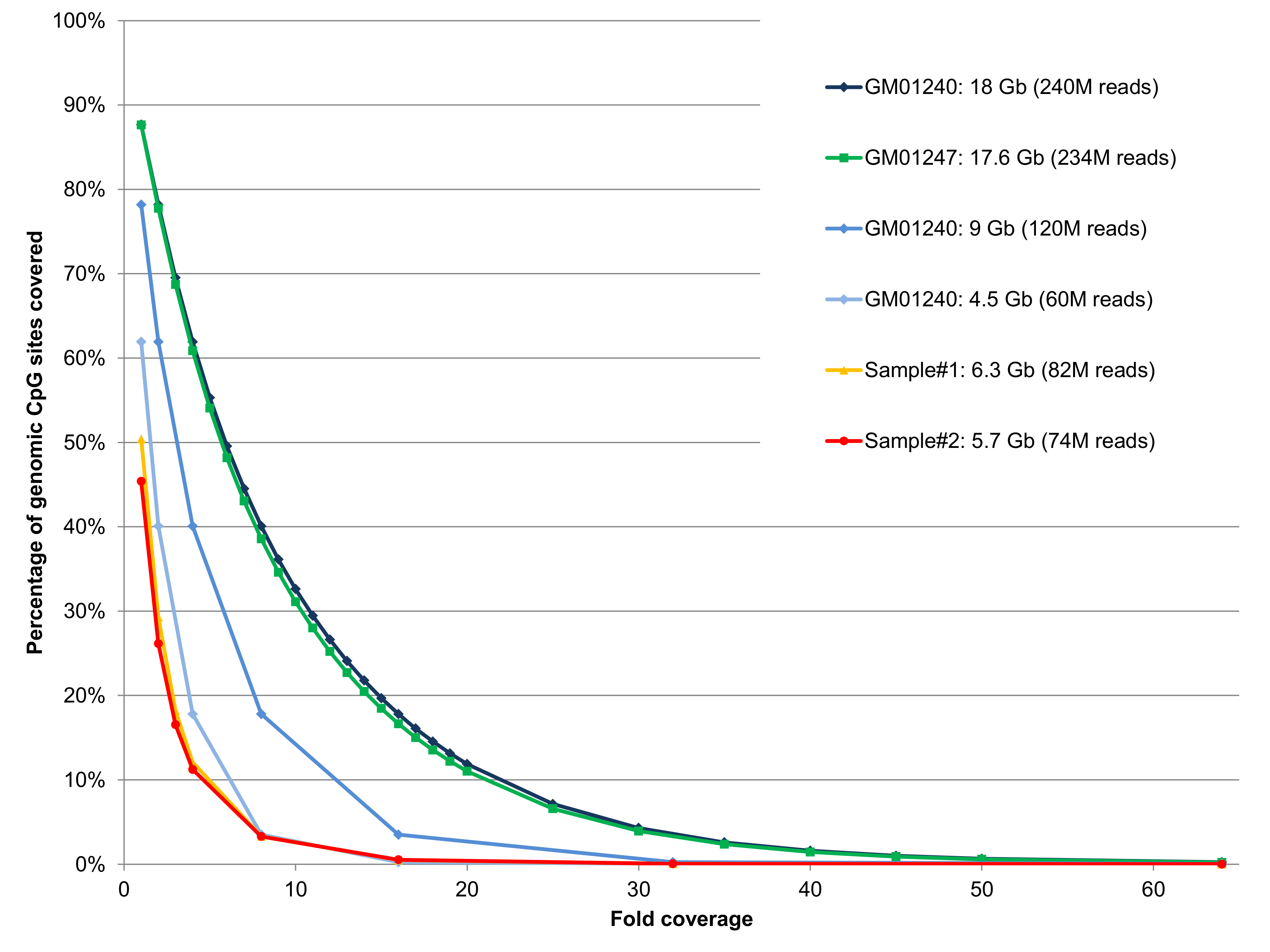

Supplement: Figure S3 — Sequencing depth and CpG coverage. The percentage of genomic CpG sites covered at different depths of sequence (fold coverage) is shown for GM01240 (240 M reads, 18 Gb of sequence ) in dark blue; for GM01247 (234 M reads, 17.6 Gb ) in green; for GM01240 (120 M reads, 9 Gb ) in blue; for GM01240 ( 60 M reads, 4.5 Gb) in light blue; for Sample#1 (82 M reads, 6.3 Gb ) in yellow; for Sample#2 ( 74 M reads, 5.7 Gb ) in red. It is important to note that Sample#1 and Sample#2 (unrelated to this study but processed in the same way) were both sequenced on one lane of an Illumina GAII resulting in a lower sequencing yield compared to GM01240 and GM01247 which were both sequenced on one lane of an Illumina HiSeq 2000. The data for the 120 M and 60 M reads graphs for GM01240 were calculated from the 240 M dataset by taking a subset of the reads equivalent to half (1/2) and a quarter (1/4) of the original dataset. All experimental datasets were sequenced to saturation according to the MEDIPS saturation analysis (data for GM01240 and GM01247 are shown on Figure S2). (TIF) [file pone.0050233.s003.tif]

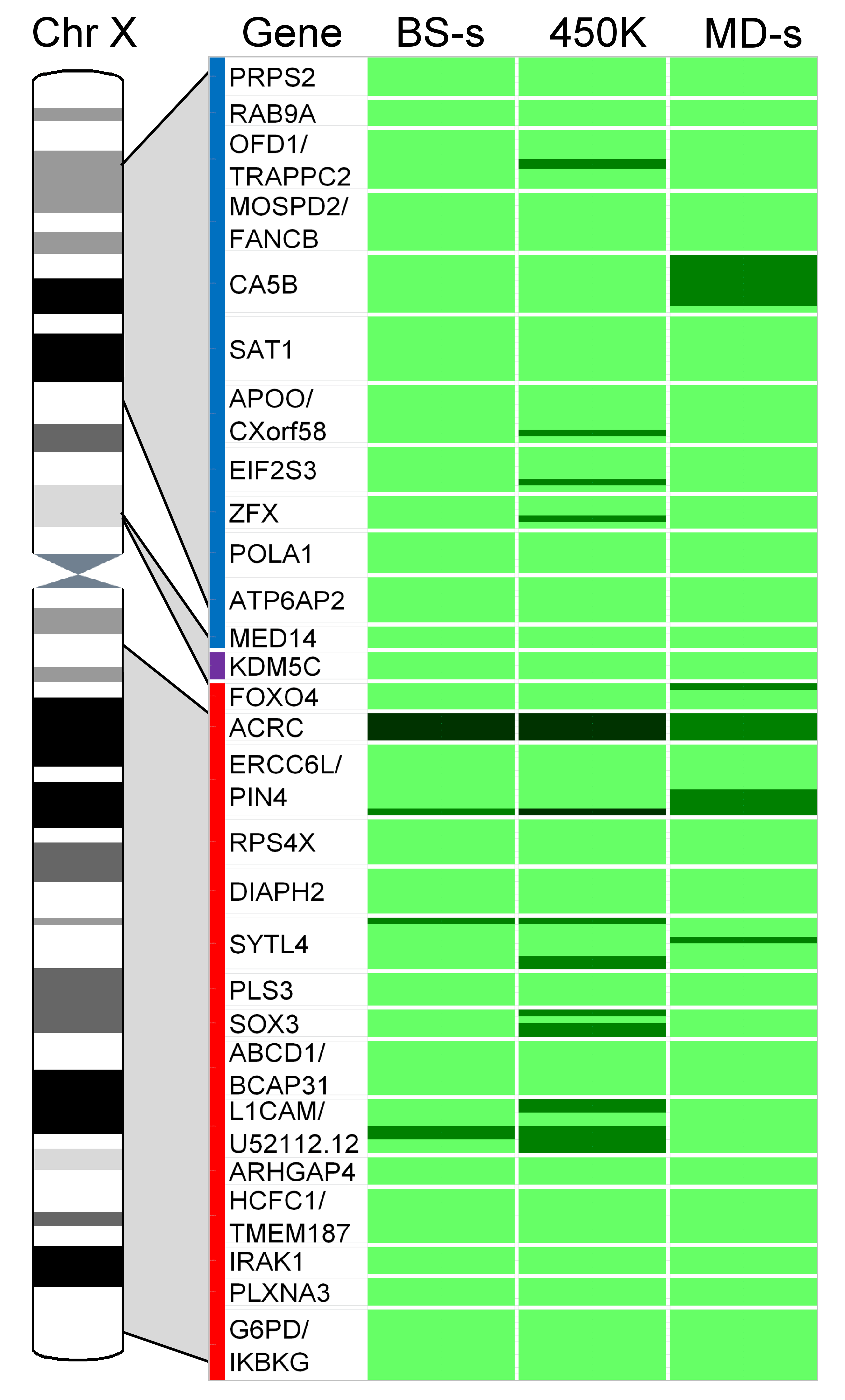

Supplement: Figure S4 — Comparison of methylation level estimates for the bisulfite sequencing (BS-s), HumanMethylation 450K (450K) and MeDIP-seq (MD-s) data. Data are shown for the 28 islands (associated with 36 genes) containing CpG sites that overlapped with those interrogated by HumanMethylation 450K array for sample GM01247. Evolutionary strata information is shown to the right of the ideogram of the human X chromosome [66]: the blue line represents the S3 stratum; the purple line represents the S2 stratum and the red line the S1 stratum. Both names are given for genes sharing a CpG island separated by “/”. Methylation level estimates for each of the techniques are shown to the right of the gene names in light green (low), green (medium), and dark green (high). (TIF) [file pone.0050233.s004.tif]
