# Supplementary material for: A Comparison of the Whole Genome Approach of MeDIP-Seq to the Targeted Approach of the Infinium HumanMethylation450 BeadChip® for Methylome Profiling
Source: PLoS One. 2012 Nov 29;7(11):e50233. doi: 10.1371/journal.pone.0050233 (PMC3510246; doi:10.1371/journal.pone.0050233)
Supplement: Table S1 — Coverage by MeDIP-seq and the HumanMethylation 450K BeadChip of different genomic features. The theoretical maximum number of sites for different features of the genome (Sites or features in the genome) was calculated as follows: The coordinates for CpG islands, regulatory elements, RefSeq genes (and other related elements) and human repetitive elements were downloaded from the ENSEMBL database (v63). CpG island shores were calculated at 2 kb either side of an island and CpG shelves as the 2 kb extending from the shores. The number of CpG shelves is fewer than the number of CpG shores because if two CpG islands are less than 4 kb apart they will be separated by one or two shores but no shelves. Coordinates for GENCODE ECRs were calculated from the GENCODE database (v8) by collapsing all overlapping GENCODE genes into expressed cluster regions (see Supporting Information). Coordinates for all CpN sites were extracted from the GRCh37 1000 Genomes reference genome (ftp://ftp.1000genomes.ebi.ac.uk/vol1/ftp/technical/reference/human_g1k_v37.fasta.gz). The coverage shown for the HumanMethylation 450K (Sites covered by design) is based on the array design and reported as the number of regions or features with at least one probe present on the array mapping to them. The following column gives the percentage of the theoretical maximum number of sites covered for each feature by the HumanMethylation 450K. For MeDIP-seq, the region or feature was defined as being covered if any part of the region or feature was covered by or overlapped with one or more sequencing reads with a mapping quality score ≥10. The number of sites covered for each sample (GM01240 (XX) or GM01247 (XY)) is given (Sites covered by ≥1 reads) as well as the percentage of the theoretical maximum number of sites covered for each feature (Percentage of genomic sites covered). (DOCX) [file pone.0050233.s005.docx]

**Table S1: Coverage by MeDIP-seq and the HumanMethylation 450K of different genomic features.**

|  |  | HumanMethylation 450K | | MeDIP-seq | | | |
| --- | --- | --- | --- | --- | --- | --- | --- |
|  |  |  |  | GM01240 | | GM01247 | |
| Feature | Sites or features in the genome | Sites covered by design | Percentage of genomic sites covered | Sites covered by ≥1 reads | Percentage of genomic sites covered | Sites covered by ≥ 1 reads | Percentage of genomic sites covered |
| CpG sites | 28162537 | 482421 | 1.7% | 24688534 | 87.7% | 24680779 | 87.6% |
| CpA sites | 207350708 | 3081 | 0.0% | 167727044 | 80.9% | 165655906 | 79.9% |
| CpT sites | 199988203 | 10 | 0.0% | 163149688 | 81.6% | 161259874 | 80.6% |
| CpC sites | 148887078 | 0 | 0.0% | 123786323 | 83.1% | 122768463 | 82.5% |
| CpG islands | 22371 | 19738 | 88.2% | 21716 | 97.1% | 21780 | 97.4% |
| CpG shores | 42731 | 34483 | 80.7% | 42175 | 98.7% | 42208 | 98.8% |
| CpG shelves | 39045 | 27898 | 71.5% | 38473 | 98.5% | 38531 | 98.7% |
| GENCODE ECRs | 310060 | 53652 | 17.3% | 288259 | 93.0% | 288725 | 93.1% |
| GENCODE ECRs ± 1 kb | 138440 | 52510 | 37.9% | 136750 | 98.8% | 137213 | 99.1% |
| Regulatory elements | 445282 | 75470 | 16.9% | 437791 | 98.3% | 437766 | 98.3% |
| RefSeq genes | 22201 | 20755 | 93.5% | 21873 | 98.5% | 21928 | 98.8% |
| RefSeq 3' UTR regions | 20179 | 13330 | 66.1% | 19420 | 96.2% | 19428 | 96.3% |
| RefSeq 5' UTR regions | 19476 | 14378 | 73.8% | 15859 | 81.4% | 16019 | 82.2% |
| RefSeq genes, first exon | 22201 | 16055 | 72.3% | 17950 | 80.9% | 18152 | 81.8% |
| RefSeq genes, gene body | 20039 | 18837 | 94.0% | 19795 | 98.8% | 19838 | 99.0% |
| RefSeq gene upstream | 22201 | 19964 | 89.9% | 21614 | 97.4% | 21685 | 97.7% |
| Repetitive elements | 8736248 | 77486 | 0.9% | 8003776 | 91.6% | 7960261 | 91.1% |
| DNA repeat elements | 3388666 | 11005 | 0.3% | 2963430 | 87.5% | 2931075 | 86.5% |
| LINEs | 1471552 | 17377 | 1.2% | 1408845 | 95.7% | 1404693 | 95.5% |
| SINEs | 1757674 | 21549 | 1.2% | 1693893 | 96.4% | 1691892 | 96.3% |
| LTRs | 693621 | 13199 | 1.9% | 665245 | 95.9% | 666255 | 96.1% |
| RNA repeats | 1405623 | 12901 | 0.9% | 1255793 | 89.3% | 1248899 | 88.9% |
| Satellite repeats | 6289 | 558 | 8.9% | 4427 | 70.4% | 5302 | 84.3% |
| Other repeats | 5718 | 810 | 14.2% | 5462 | 95.5% | 5488 | 96.0% |
| Unknown repeats | 7105 | 87 | 1.2% | 6681 | 94.0% | 6657 | 93.7% |

The theoretical maximum number of sites for different features of the genome (Sites or features in the genome) was calculated as follows: The coordinates for CpG islands, regulatory elements, RefSeq genes (and other related elements) and human repetitive elements were downloaded from the ENSEMBL database (v63). CpG island shores were calculated at 2 kb either side of an island and CpG shelves as the 2 kb extending from the shores. The number of CpG shelves is fewer than the number of CpG shores because if two CpG islands are less than 4 kb apart they will be separated by one or two shores but no shelves. Coordinates for GENCODE ECRs were calculated from the GENCODE database (v8) by collapsing all overlapping GENCODE genes into expressed cluster regions (see Supporting Information). Coordinates for all CpN sites were extracted from the GRCh37 1000 Genomes reference genome (ftp://ftp.1000genomes.ebi.ac.uk/vol1/ftp/technical/reference/human_g1k_v37.fasta.gz).

The coverage shown for the HumanMethylation 450K (Sites covered by design) is based on the array design and reported as the number of regions or features with at least one probe present on the array mapping to them. The following column gives the percentage of the theoretical maximum number of sites covered for each feature by the HumanMethylation 450K.

For MeDIP-seq, the region or feature was defined as being covered if any part of the region or feature was covered by or overlapped with one or more sequencing reads with a mapping quality score ≥ 10. The number of sites covered for each sample (GM01240 (XX) or GM01247 (XY)) is given (Sites covered by ≥1 reads) as well as the percentage of the theoretical maximum number of sites covered for each feature (Percentage of genomic sites covered).
